# Two Strategies for Response to 14°C Cold-Water Immersion: Is there a Difference in the Response of Motor, Cognitive, Immune and Stress Markers?

**DOI:** 10.1371/journal.pone.0109020

**Published:** 2014-10-02

**Authors:** Marius Brazaitis, Nerijus Eimantas, Laura Daniuseviciute, Dalia Mickeviciene, Rasa Steponaviciute, Albertas Skurvydas

**Affiliations:** 1 Sports Science and Innovation Institute, Lithuanian Sports University, Kaunas, Lithuania; 2 Department of Educational Studies, Kaunas University of Technology, Kaunas, Lithuania; 3 Department of Laboratory Medicines, Medical Academy, Lithuanian University of Health Science, Kaunas, Lithuania; Technion - Israel Institute of Technology, Israel

## Abstract

Here, we address the question of why some people have a greater chance of surviving and/or better resistance to cold-related-injuries in prolonged exposure to acute cold environments than do others, despite similar physical characteristics. The main aim of this study was to compare physiological and psychological reactions between people who exhibited fast cooling (FC; n = 20) or slow cooling (SC; n = 20) responses to cold water immersion. Individuals in whom the T_re_ decreased to a set point of 35.5°C before the end of the 170-min cooling time were indicated as the FC group; individuals in whom the T_re_ did not decrease to the set point of 35.5°C before the end of the 170-min cooling time were classified as the SC group. Cold stress was induced using intermittent immersion in bath water at 14°C. Motor (spinal and supraspinal reflexes, voluntary and electrically induced skeletal muscle contraction force) and cognitive (executive function, short term memory, short term spatial recognition) performance, immune variables (neutrophils, leucocytes, lymphocytes, monocytes, IL-6, TNF-α), markers of hypothalamic–pituitary–adrenal axis activity (cortisol, corticosterone) and autonomic nervous system activity (epinephrine, norepinephrine) were monitored. The data obtained in this study suggest that the response of the FC group to cooling vs the SC group response was more likely an insulative–hypothermic response and that the SC vs the FC group displayed a metabolic–insulative response. The observations that an exposure time to 14°C cold water—which was nearly twice as short (96-min vs 170-min) with a greater rectal temperature decrease (35.5°C vs 36.2°C) in the FC group compared with the SC group—induces similar responses of motor, cognitive, and blood stress markers were novel. The most important finding is that subjects with a lower cold-strain-index (SC group) showed stimulation of some markers of innate immunity and suppression of markers of specific immunity.

## Introduction

It is well established that exposure to an acute cold stress: increases the levels of catecholamine [61], [46], [60] and other stress hormones [61], [49], [33], [60]; impairs vigilance, overall mood [35], motor and cognitive performance [59], [18], [35], [51], [7], and working memory [19], [54]; increases spinal reflex excitability [14], [47]; and suppresses [58], [33] or stimulates [27], [8], [13], [66] the immune system. These are physiological characteristics that are of critical importance for the survival, health, and well-being of humans who are exposed to occupational and/or recreational extreme-cold conditions. Several individual factors, such as age, sex, body composition, exercise, acute and chronic diet, genotype, fitness level, and health, may modify the body's responses to cold [32], [38]. The pattern of cold responses is dependent on the type, intensity and duration of the cold exposure [31], [32], [38]. For instance, very mild decreases in core temperature (∼0.5°C) have little or even stimulatory effects on immune function [8], but modest (∼1°C) decreases in core temperature have depressive effects on immune function [12]. Immune function seems to be stimulated by acute stress but is suppressed by chronic stress [20], [67], [53], [34]. This dual response can be beneficial for some types of immune responses but deleterious for others.

Here, we induced cold stress by intermittent body immersion in cold water and examined the responses in two physically similar groups of subjects: those who exhibited a fast decrease in rectal temperature (T_re_) (fast cooling, FC) and those who exhibited a slow decrease in T_re_ (slow cooling, SC). Findings may address the question of why some people have a greater chance of surviving and/or better resistance to cold-related injuries in prolonged exposure to acute cold environments than do others, despite similar physical characteristics. The origin of thermoregulation in non-cold-adapted humans involves metabolic (an increase in heat production), hypothermic (a reduction in T_re_), and insulative (a reduction in T_sk_ relative to T_re_) patterns of physiological responses to exposure to acute cold. It appears that a response to cold exposure is mainly expressed by a proportional change in the magnitude of the physiological response of the three pattern types [38], [5]. Based on this assumption, we reasoned that, originally, FC vs SC relates more to an insulative–hypothermic response to cold, and that SC vs FC relates more to a metabolic–insulative response to cold. We have not found any data in the literature that indicate a difference in responses of motor and cognitive performance, markers of stress, and immune system between physically similar subjects who originally exhibited two different response strategies (FC vs SC) to acute cold stress (i.e., 14°C cold-water immersion). There is no doubt that acute stress (including cold stress) evokes majority of physiological processes in the human body (for instance, it induces neuroendocrinological, psychoneuroimmunological responses) and as a consequence alters psychological/cognitive and motor functioning [40]. In the current study we investigated the effect of acute cold stress on physiological and psychological characteristics and their possible complex-interaction in two cold tolerance groups (which we believe was studied here for the first time) and that is of critical importance in an occupational hazard to accidental hypothermia.

T_re_ decreased faster during cooling in the FC group (insulative–hypothermic response) compared with the SC group (metabolic–insulative response). We reasoned that the FC group would therefore perceive greater cold stress, which would be reflected in larger increases in the cold strain index (CSI) (see “Cold strain index” section) and would have greater effects than SC on stress markers (cortisol, corticosterone, epinephrine and norepinephrine concentrations), and in markers of immune function (neutrophils, leucocytes, lymphocytes, monocytes, interleukin 6 (IL-6) and tumour necrosis factor-α (TNF-α) concentrations). We also expected that major cold stress induced by FC would have greater effects than SC on motor (spinal reflexes, supraspinal reflexes and voluntary and electrically induced skeletal muscle contraction force) and cognitive (unpredictable task switching (executive function), the forward digit-span task (short term memory), and the forced-choice recognition memory test (short term spatial recognition) performance.

## Methods

### Rationale for experiment

The experiment was designed to explore the effect of whole-body acute cold stress on physiological and psychological markers measured between two groups of subjects who were similar in physical characteristics (Table 1) but who originally exhibited two different response strategies to 14°C cold-water immersion (i.e., fast cooling vs slow cooling). Individuals in whom the T_re_ decreased to a set point of 35.5°C before the end of the 170-min cooling time were indicated as the FC group; individuals in whom the T_re_ did not decrease to the set point of 35.5°C before the end of the 170-min cooling time were classified as the SC group. The set points (i.e., T_re_; cooling time) are relevant to a moderate survival risk zone in cold water at 14°C in humans (based on the classic curve for Cold Water Survival introduced by the Canadian Red Cross).

**Table 1:** Physical characteristics of subjects.

	FC	SC
Age, yr	21.2±1.1	22.3±1.7
Height, cm	184.0±5.1	183.3±5.8
Mass, kg	77.5±5.9	79.1±8.3
Body surface area, m^2^	2.02±0.03	2.00±0.01
Mean subcutaneous fat, mm	10.4±2.9	11.8±4.1
Body mass index, kg/m^2^	22.8±3.1	21.6±3.9
Body fat, %	14.6±4.2	16.9±5.5

Fast cooling group (FC); Slow cooling group (SC). Values are means ± SD.

### Participants and ethical approval

Forty healthy young men were recruited from a larger ongoing association study based on their tolerance to cold exposure. From the earlier study, we selected an FC group, whose T_re_ decreased to the set point of 35.5°C before the end of the 170-min cooling time, and an SC group, who's T_re_ did not decrease to the set point of 35.5°C before the end of the 170-min cooling time. The two groups comprised 20 subjects each. This was intended to provide the largest power to detect differences between the cold tolerance groups.

The physical characteristics of the subjects are presented in Table 1. The subject's weight (in kg), body fat (in percent) (TBF-300 body composition analyser, Tanita, UK Ltd., West Drayton, UK) and height (in cm) were measured, and body mass index (BMI) was calculated. Body surface area (in m^2^) was estimated as [63].
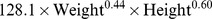



Skinfold thickness (in mm) was measured (SH5020 skinfold calliper, Saehan, Masan, Korea) at 10 sites (chin, subscapular, chest, side, suprailium, abdomen, triceps, thigh, knee and calf [39]; and the mean subcutaneous fat thickness was calculated [2]. There was no significant difference in body surface area, mean subcutaneous fat and BMI between the FC and SC groups (P>0.05).

The subjects were moderately physically active (<2 h/week) but did not participate in any formal physical exercise or sport programme. They had not been involved in any temperature-manipulation programme or extreme-temperature exposure for at least 3 months. Subjects with any existing medical condition or taking medication that could affect natural thermoregulation were excluded from the study. Subjects were also asked to refrain from taking any medication during the study.

Each subject volunteered to participate after being informed of the purpose, experimental procedures and known risks of the study. Each subject read and signed a written informed consent form consistent with the principles outlined in the Declaration of Helsinki. The Kaunas Regional Research Ethics Committee approved this study (No. BE-2-41).

### Familiarization session and experimental protocol

The study comprised a familiarization trial and an experimental trial. To attain a stable level of performance, at least 4 days before the experimental trial, participants attended a familiarization session during which they were introduced to the experimental procedures for cognitive and neuromuscular testing. During this session, each subject performed all three cognitive tests (Unpredictable task switching, the forward digit-span task, the forced-choice recognition memory test) (see “Cognitive performance” section) and learned to perform them correctly in the same manner as it was required for the experimental session. For neuromuscular testing the subjects learned to achieve and maintain maximal-effort ankle plantar flexion for 3 to 4 s with a 250-ms stimulation train test at 100 Hz (TT-100 Hz) superimposed on a voluntary contraction and the tolerance to electrical stimulation was assessed. To control for circadian fluctuations in blood stress hormones and body temperature, the experiment began at 7.30 am. Subjects were instructed to sleep for >8 h the night before the experiment and to refrain from alcohol, heavy exercise and caffeine for at least 24 h before the experiment. The subjects refrained from consuming any food for at least 12 h before the experiment. To standardize the state of hydration and the feeling of thirst, subjects were allowed to drink still water as desired until 60 min before the experiment. The experiments were performed at 22°C (room temperature) and 60% relative humidity.

**Figure 1:**
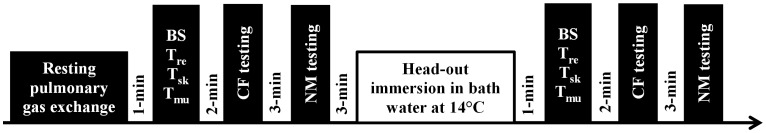
Research design. BS – blood samples, T_re_ – rectal temperature, T_mu_ – muscle temperature, T_sk_ – skin temperature. Cognitive function (CF) testing involved the unpredictable task switching test (executive function), the forward digit-span task test (short term memory), and the forced-choice recognition memory test (short term spatial recognition). Neuromuscular (NM) testing involved evaluation of spinal (H-reflexes, M-waves) and supraspinal (V-waves) excitability, evaluation of muscle contractility characteristics induced by a 1-s electrical stimulation at 1 Hz, 20 Hz, 100 Hz and TT-100 Hz; evaluation of maximal voluntary contraction torque and central activation of exercising muscle was performed with a TT-100 Hz superimposed stimulation on the maximal voluntary contraction. Intermittent head-out immersion in bath water at 14°C continued until the rectal temperature decreased to 35.5°C or until 170 min total (120 min maximum total immersion time), at which time the immersion ended regardless of the rectal temperature.

On arrival at the laboratory, the subject was asked to rest in a semi-recumbent posture for 10 min dressed in a T-shirt, swim shorts and socks. The resting pulmonary gas exchange for the next 20 min was recorded in the same semi-recumbent posture. T_sk_, muscle (T_mu_) and T_re_ temperature stabilizations were assessed, and control measurements of T_sk_, T_mu_ and T_re_ were made (Fig. 1). Following this, a blood sample was drawn from a vein and was stored for later analysis. The subject was then seated at a table, and cognitive testing was performed. Upon completion of the cognitive tests, the subject was positioned in the dynamometer chair, stimulating electrodes were placed over the tibial nerve and electromyography electrodes were placed over the soleus (SOL) muscle of the right leg, and the reflexes were assessed. After a 5-min rest, during which the electrodes were moved from over the tibial nerve to over the posterior calf muscle, the force-generating capacity of the posterior calf muscle was assessed by applying 1-s trains of electrical stimuli at 1, 20, TT-100 Hz and 100 Hz. About 3 s was needed to change the stimulation frequency. After a 1-min rest, two attempts of a 3 to 4 s maximal voluntary contraction (MVC) interspaced with a 1-min rest interval was performed with a TT-100 Hz superimposed on the voluntary contraction. These TT-100 Hz contractions were used to assess the central activation ratio (CAR) of the posterior calf muscle. The CAR (in percent) was calculated as the ratio of the maximal voluntary force to the peak force generated with the additional TT-100 Hz superimposed on an MVC.

After the muscle testing, the subject began the intermittent water immersion cooling protocol. It is suggested that intermittent cooling represents a more efficient way of removing body heat than compare to continue cooling [11]. During cooling, every 20 min, the subject was asked to step out of the bath and rest for 10 min in the room environment, and then to return to the water bath for the next 20 min of immersion. The water bath temperature was 14°C and a head-out immersion procedure was used. The procedure continued until the T_re_ decreased to 35.5°C or until 170 min total (120 min maximum total immersion time), at which time the immersion ended regardless of the T_re_. The exposure time until the T_re_ achievement was recorded. During immersion and the rest intervals, the subject remained in a semi-recumbent posture with his arms folded across the chest and with the legs almost straight and together. Ratings of subjective thermal and shivering sensation and T_re_ were recorded every 5 min throughout the cooling procedure. Pulmonary gas exchange was recorded only during each 20-min water immersion. Within ∼1 min after leaving the bath, the volunteer was towel dried, T_sk_ and T_mu_ were measured, and a blood sample was taken. After the end of the cooling protocol, cognitive and neuromuscular testing was performed in the same order as before the cold water immersion.

### Body temperature measurements

T_re_ was measured throughout the experimental trial using a thermocouple (Rectal Probe, Ellab, Hvidovre, Denmark; accuracy ±0.01°C) inserted to a depth of 12 cm past the anal sphincter. The rectal thermistor sensor was placed by each subject. T_mu_ and T_sk_ were measured before and at the end of the water immersion. The T_mu_ was measured with a needle microprobe (MKA, Ellab) inserted ∼3 cm under the skin covering the lateral gastrocnemius muscle of the right leg. The skin was prepared before each intramuscular temperature measurement by shaving and disinfecting before and after insertion with a cotton-wool tampon soaked with medicinal alcohol. After the first measurement, the insertion area was marked with a 0.5-cm-diameter circle. This was done to ensure repeatability and to allow use of the same insertion point for each measurement (i.e., before and after cooling). T_sk_ was measured with thermistors taped at three sites: back, thigh and forearm (DM852, Ellab; accuracy ±0.01°C), and mean T_sk_ was calculated by the Burton [9] equation as: 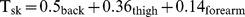



### Cold strain index (CSI)

The CSI was based on the core temperature (T_re_) and mean T_sk_ as a rating of cold strain on a universal scale of 0 to 10 as 1 to 2 (no/little cold strain), 3 to 4 (low cold strain), 5 to 6 (moderate cold strain), 7 to 8 (high cold strain) and 9 to 10 (very high cold strain). The CSI was calculated as follows [43]:
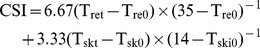



The CSI was calculated before (T_re0_, T_sk0_) and at the end of passive cooling (T_re*t*_, T_sk*t*_); 14°C – water temperature; 35°C – T_re_ threshold. T_re_ and T_sk_ were assigned a weighting using the constants 6.67 and 3.33, respectively.

### Measurement of subjective thermal and shivering sensation

The method to measure subjective ratings for the whole body has been described elsewhere [24], [6]. Briefly, the rating of thermal sensation ranged from 1 (very cold) to 9 (very hot), with 5 being neutral. The shivering sensation ranged from 1 (vigorously shivering) to 4 (not at all) being neutral. The rating of perception was reported by the participants every 5 min during passive cooling. A mean rating score was calculated for each session.

### Isometric torque and electrical stimulation

The isometric torque of ankle plantar flexion muscles was measured using an isokinetic dynamometer (System 4; Biodex Medical Systems, Shirley, NY, USA) calibrated according to the manufacturer's service manual with a correction for gravity performed using the Biodex Advantage program (version 4.X). Subjects were seated in the dynamometer chair with the trunk inclined at 45° with respect to the vertical, and with hip, knee and ankle joint angulations of, respectively, 90°, 100° (full knee extension  = 180°) and 90°. For MVC measurement, the subject was instructed to achieve and maintain maximal effort of ankle plantar flexion for 3 to 4 s. Each trace was inspected visually to ensure that there were no artefactual spikes at the start of the signal curve. The arms were crossed on the chest with the hands grasping the trunk-supporting belt during all tests on the dynamometer. To help ensure a maximal effort, standard vocal encouragement was provided during each voluntary ankle plantar flexion trial by the same experienced investigator. The subject was asked to perform two attempts with a rest interval of 1 min. The best attempt was recorded.

The equipment and procedure for electrically stimulated torque have been described previously [7]. Briefly, muscle stimulation was applied using flexible surface electrodes (MARP Electronic, Krakow, Poland), covered with a thin layer of electrode gel (ECG–EEG Gel; Medigel, Modi'in, Israel), with one electrode (8×12 cm) placed transversely across the width of the proximal portion of the posterior calf just below the popliteal fossa, and the other electrode (8×8 cm) covered the distal portion of the muscle just below the muscle fibres of the gastrocnemius. A constant current electrical stimulator (MG 440; Medicor, Budapest, Hungary) was used to deliver 0.5-ms square-wave pulses at 150 V. Peak torques induced by a 1-s electrical stimulation at 1 Hz (P1; representing the properties of muscle excitation–contraction coupling), at 20 Hz (P20; representing the steep section of the force–frequency relationship curve) and at 100 Hz (P100; which is close to maximal force) were measured with a 3-s rest interval between electrical stimulations. The half-relaxation time (in ms) was measured in resting TT-100 Hz contractions. The half-relaxation time (HRT) was calculated as the time taken for torque to decline from the peak value to half of that value at the end of the TT-100 Hz contraction. A rest interval of 1 min was set between the electrical stimulation trial and MVC measurements.

### Reflex recordings

The subject positioning, testing environment and electrical stimulator assessment were essentially the same as those described above. After careful preparation of the skin (shaving, abrading, and cleaning with alcohol wipes) to obtain low impedance, bipolar Ag–AgCl surface bar electrode (10 mm diameter, 20 mm centre-to-centre distance) (DataLog type no. P3X8 USB, Biometrics Ltd, Gwent, UK) were used for electromyography (EMG) recording. For the SOL, the electrode was placed over the SOL ∼13 cm above the calcaneus below the muscle fibres of the gastrocnemius. The actual electrode position was marked, and the same recording site was used in the pre- and post-session measurements. The ground electrode was positioned on the tarsus of the same leg. EMG signals were recorded by amplifiers (gain 1000), with signal measurements using a third-order filter (18 dB/octave) bandwidth of 20–460 Hz. The analogue signal was sampled and converted to digital form at a sampling frequency of 5 kHz. The EMG signal was telemetered to a receiver that contained a differential amplifier with an input impedance of 10 MΩ, the input noise level was less than 5 µV, and the common mode rejection ratio was higher than 96 dB. Before recording the EMG, we set the channel sensitivity at 3 V and excitation output at 4600 mV as recommended by the manufacturer. EMG files were stored simultaneously on the biometrics memory card and PC hardware, and dedicated software (Biometrics DataLOG, Gwent, UK) was used for data processing and analysis.

SOL H-reflexes, V-waves and M-waves were evoked by 0.5-ms square-wave pulses stimulated by a cathode placed in the popliteal cavity and an anode distal to the patella over the posterior tibial nerve with an inter-electrode distance of ∼4 cm. The resting maximal H-reflex (H_max_; reflects the efficiency of the transmission in Ia afferent-α motor neuron synapses) and maximal M-wave (M_max_; reflects sarcolemmal excitability) were obtained by increasing the electrical intensity by 3 V every 10 s in the 30–150 V range. With increasing stimulation intensity, the H-reflex response initially increased progressively before decreasing and then disappearing, whereas the M-wave achieved its maximum and remained stable. Thereafter, the subject was instructed to perform three brief MVCs of the plantar flexor muscles with at least a 1-min rest between contractions. A superimposed stimulus (at M_max_ intensity) was evoked to obtain the V-wave (V_sup_). The peak-to-peak amplitude of the V-wave reflects the magnitude of the central descending neural drive to spinal motor neurons, although spinal factors such as motor neuron excitability and pre- or post-synaptic inhibition may also be involved [1]. M-wave amplitude was also used to normalize the amplitude of the reflex waves recorded (i.e., H_max_/M_max_ ratio). This was done to ensure that any changes in the evoked H_max_ and V_sup_ amplitudes reflected changes at the muscle fibre membrane or neuromuscular junction. The latencies of the electrical evoked action potentials were calculated from the stimulation artefact at the peak of the wave.

### Spirometry

A mobile spirometry system (Oxycon Mobile, Jaeger/VIASYS Healthcare, Hoechberg, Germany) was used to measure pulmonary gas exchange at rest and during water immersion. This system uses a tightly fitting face mask that covers the nose and mouth with a lightweight integrated flow meter (Triple V volume sensor; 45 g) with a dead space of 30 ml. The system monitors ventilatory parameters, oxygen uptake and VCO_2_ production on a breath-by-breath basis. The processing, recording and battery system comprises two units attached to a belt, which was hung as close as possible to the subject's nose and mouth during immersion. The data were stored on memory cards and PC hardware. This instrument was calibrated before recording as indicated in the manufacturer's manual by using the automatic volume- and gas-calibration functions. A flow-volume sensor calibration procedure assures that the Oxycon quantification system (including the amplifier, Triple V sensors and pressure transducer) is functioning correctly. The gas analyser and delay time calibrations were also automatic, as provided by the manufacturer: a calibration gas at 180 kPa (15.2% O_2_, 5.02% CO_2_ and 79.78% N_2_) was introduced to the Oxycon to attain gain, offset and delay times within 1%. Oxygen consumption was recorded in 5-s increments. As recommended, the data collected during each first 5 min of each 20-min immersion were not used in any calculations because of reflex hyperventilation caused by cold water immersion. Because the total water-immersion time differed between subjects, the mean values for VO_2_ (in ml/min/kg and l/min) and VCO_2_ (l/min) were calculated.

Metabolic heat production (MHP) was calculated in W from the respiratory gas exchange measurements of VO_2_ (l/min) and the respiratory exchange ratio (RER = VCO_2_÷VO_2_) according to Peronnet and Massicotte [48] as follows:
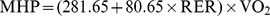



Because the sites of body temperature measurements reflect the core and skin temperatures, predictions of shivering (M_shiv_) (in W/m^2^) are based largely on data from these sites. A predictive formula to estimate metabolic rate due to shivering metabolism has been proposed as follows [62]:
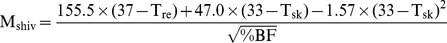
where %BF is the percentage of body fatness; 37°C – T_re_ set point; 33°C – T_sk_ set point. M_shiv_ was calculated from the values measured at the end of immersion when T_re_ and T_sk_ were the lowest.

### Cognitive performance

It has been well documented that sufferers from blood-injection-injury phobia often experience emotional fainting (vasovagal syncope), display emotional disgust, excessive fear and avoidance behavior [3], [26]. None of these blood-injection-injury phobia markers were observed in our subjects during needle insertion and/or blood sampling procedure.

A programmed cognitive test battery was used to assess executive function, short-term memory, and visual recognition memory. All tasks were computer controlled, and the information was presented on the screen of a laptop (HP Compaq 6730b). In a pilot study, participants (n = 53) completed the cognitive test battery on four separate occasions, and the performance on the three tests was compared using the intra-class test of reliability by analysis of variance (ANOVA) [4]. The reliability was considered acceptable because the intra-class correlations were R≥0.80 for all tests and there were no significant differences in performance. The coefficient of variation for repeated tests was <5%. All tests were performed in a quiet and semi-darkened laboratory with a laptop screen ∼40 cm in front of the participant. The test battery took ∼10 min to perform and included the following tasks.

*Unpredictable task switching*: The *odd/even* test [44] measures choice reaction time (in ms) to an unpredictable digit-choice protocol. This test reflects cognitive flexibility (executive function), which is defined as the ability to adjust to changing demands [16]. Forty randomized single-digit stimuli from 0 to 9 of 180 s duration were displayed with varying inter-stimulus intervals in the middle of the screen. As fast as possible, the subject had to press the button for the even (right button) or odd (left button) digit corresponding to the digit presented.

In a highly cited article, the psychologist Miller [42] suggested that normal adult short-term memory has a forward memory span of about seven items plus or minus two. The *forward digit-span task* [42], [16] tests the ability to remember a sequence of digits in a short time. This test assesses short-term memory [16], which can hold a limited amount of information in a very accessible state temporarily. The subject was instructed to remember a seven-digit sequence displayed for 3 s in the middle of the screen. The subject then immediately entered the digits using a numeric keyboard in the same consecutive sequence as presented. If the digits were identified correctly, for the next attempt, the sequence was one digit longer; if an error was made, the next sequence was one digit shorter. There were 16 sequences. The mean number of digits identified successfully was recorded.

The *forced-choice recognition memory test* [52] assesses visual recognition memory. After looking at nine visual figures displayed for 15 s in the middle of the screen, the subject was required to recognize the nine items from 28 figures presented in the study list in any order. The number of correctly identified images was recorded.

### Blood variables

Blood samples were collected by venepuncture into vacuum tubes (EDTA-K3, 3 ml) before and after cold water immersion, mixed gently by inverting 8–10 times, and kept at room temperature until analysed for differential blood cell counting of neutrophils, leucocytes, lymphocytes and monocytes. Blood samples were analysed 1 to 2 h after blood collection using an automated haematology analyser (XE-5000, Sysmex Corp., Kobe, Japan).

Blood samples for measurement of epinephrine and norepinephrine concentrations were collected in vacuum tubes using EDTA as an anticoagulant (EDTA-K3, 3 ml), mixed gently by inverting 8–10 times and kept at 2–8°C until centrifugation. Blood samples were centrifuged at 1200×*g* for 15 min within 30 min of blood collection. Plasma samples were separated as soon as possible (maximum 10–15 min) from the red cells after centrifugation, and kept at −70°C until analysis. Epinephrine and norepinephrine concentrations were measured using an ELISA kit (Gemini analyser, Stratec Biomedical GmbH, Birkenfeld, Germany).

Blood samples for measurement of total cortisol, corticosterone, IL-6 and TNF-α concentrations were collected by venepuncture into vacuum tubes for serum separation using a gel separator (5 ml). Blood samples were taken in the morning time (8 a.m.) that is about 1-h after awakening, when cortisol concentration level in the blood was expected to be close to the peak of the natural cortisol cycle [58]. Blood samples were allowed to clot, and the serum was separated by centrifugation at 1200×*g* for 15 min. The serum samples were aliquoted and stored at −70°C until analysis. Concentrations of corticosterone, IL-6 and TNF-α were measured by ELISA (Gemini analyser, Stratec Biomedical), and cortisol concentration was measured using an automated enzyme immunoassay analyser, AIA-2000 (Tosoh Corp., Tokyo, Japan).

### Calculations and statistical analysis

The data were tested for normal distribution using the Kolmogorov–Smirnov test, and all scale data were normally distributed. Statistical analysis involved general linear model analysis of variance (ANOVA) for repeated measures with FC and SC as a between-group factor and time as within-group factor of two levels (before and after cooling) on dependent variables (body temperatures, pulmonary gas exchange, MHP, reflexes, blood variables, and motor and cognitive performance). Physical characteristics of subjects (Table 1) and changes in CSI and M_shiv_ were analyzed via one-way analysis of variance with FC vs SC as a between-group factor. For all ordinal data the nonparametric Wilcoxon signed-rank test was used to compare changes in subjective ratings of perceptions (thermal and shivering sensations). Pearson correlation coefficients were used to identify relationships between variables. Descriptive data are presented as mean and standard deviation (SD). Observed power (OP) was calculated for all mechanical indicators based on an alpha level of 0.05, sample size (n = 20), standard deviation and average level of variables. The level of significance was set at p<0.05 and all statistical analyses were performed using IBM SPSS Statistics 22 (IBM Corporation, Armonk, NY).

## Results

### Changes in rectal, muscle and skin temperatures

In the FC group, the time to cool the body from a T_re_ of 37.0±0.3°C before cooling to 35.5°C (*F*_1,38_ = 670.78, P<0.001, OP>99%) was 96.3±37.7 min (Table 2), and the time to cool the body to 35.5°C varied in the range between 40 and 150 min. In the SC group, the time to cool the body from a T_re_ of 37.1±0.2°C to 36.2±0.41°C (*F*_1,38_ = 93.50, P<0.001, OP>99%) (time × group interaction, *F*_1,38_ = 21.63, P<0.001 between groups, OP>99%) was 170 min (*F*_1,38_ = 81.88, P<0.001 between groups, OP>99%), and the T_re_ at the end of the 170-min cooling time varied in the range from 35.7°C to 36.6°C. In the FC group, T_mu_ decreased during cooling by 6.9±2.3°C from 36.6±0.4°C before cooling (*F*_1,38_ = 175.89, P<0.001, OP>99%). In the SC group, T_mu_ decreased during cooling by 6.2±2.5°C from 36.4±0.4°C before cooling (*F*_1,38_ = 118.48, P<0.001, OP>99%) (*F*_1.00_ = 0.69, P>0.05 between groups, OP<15%). In the FC group, T_sk_ decreased during cooling by 13.1±1.8°C from 32.3±0.6°C before cooling (*F*_1,38_ = 802.51, P<0.001, OP>99). In the SC group, T_sk_ decreased during cooling by 13.7±1.5°C from 32.3±0.7°C before cooling (*F*_1,38_ = 1409.12, P<0.001, OP>99%) (*F*_1,38_ = 1.73, P>0.05 between groups, OP<25%).

**Table 2:** Body temperatures before and after body cooling.

	FC		SC	
	Before	After	Before	After
T_re_, °C	37.0±0.3	35.5 (fixed)* #	37.1±0.2	36.2±0.4*
T_mu_, °C	36.6±0.4	29.7±2.1*	36.4±0.4	30.2±2.5*
T_sk_, °C	32.3±0.6	19.3±1.8*	32.3±0.7	18.4±1.5*

*P<0.05, compared with before;

# P<0.05 between fast cooling (FC) and slow cooling (SC) groups. T_re_ – rectal temperature, T_mu_ – muscle temperature, T_sk_ – skin temperature. Values are means ± SD.

### VO_2_ and MHP

The change in VO_2_ and MHP from before to after body cooling did not differ between the FC and SC groups. However, metabolic shivering was significantly greater after cooling in the FC group than in the SC group (*F*_1,38_ = 7.17, P<0.05 between groups, OP>70%) (Table 3).

**Table 3:** VO_2_, metabolic heat production and metabolic shivering before and during body cooling.

	FC		SC	
	Before	After	Before	After
VO_2_, ml/min/kg	3.8±0.5	13.1±3.9*	3.9±0.73	13.5±3.6*
Metabolic heat production, W/m^2^	24.7±6.5	150.8±52.3*	26.3±10.8	157.6±47.8*
Metabolic shivering, W/m^2^		81.6±14.3#		60.1±12.1

*P<0.05, compared with before;

# P<0.05 between fast cooling (FC) and slow cooling (SC) groups. Values are means ± SD.

### CSI and subjective ratings of thermal and shivering sensation

During cooling, the changes in subjective sensations did not differ significantly between the FC and SC groups (Table 4). The CSI after cooling was 7.4±0.28 (high cold strain) and 5.6±1.3 (moderate cold strain) in the FC and SC groups, respectively (*F*_1,38_ = 26.11, P<0.001 between groups, OP>99%).

**Table 4:** Subjective sensation rating (in points) during passive body cooling.

	FC	SC
Shivering sensation	2.1±0.6	2.3±0.5 n.s.
Thermal sensation	2.7±0.5	2.8±1.8 n.s.

n.s. P>0.05, non-significant between fast cooling (FC) and slow cooling (SC) groups. Values are means ± SD.

### Skeletal muscle force-generating capacity

The FC and SC groups did not differ significantly on torque during electrically induced contractions, MVC, CAR and HRT (Table 5). The effect of cooling was significant for P100 (main time effect, *F*_1,38_ = 96.78, P<0.001, OP>99%), P1/P100 (main time effect, *F*_1,38_ = 83.71, P<0.001, OP>99%) and HRT (main time effect, *F*_1,38_ = 50.76, P<0.001, OP>99%); however, there were no significant differences in the changes in these variables between the FC and SC groups.

**Table 5:** Voluntary and electrically induced skeletal muscle properties before and after cooling.

	FC		SC	
	Before	After	Before	After
MVC, Nm	148.8±34.2	135.6±33.1	168.8±43.8	161.6±34.1
CAR, %	99.4±1.6	99.9±0.5	99.6±1.6	99.5±1.2
P1, Nm	16.5±5.8	17.1±6.1	20.1±5.8	19.5±4.8
P20, Nm	86.4±25.4	81.4±22.2	96.1±23.3	90.4±19.5
P100, Nm	97.8±27.1	79.8±24.6*	106.5±28.2	87.7±21.4*
P1/P100, %	16.7±2.4	21.4±3.2*	19.1±3.1	23.5±3.4*
HRT, ms	97.2±14.4	123.3±20.2*	96.1±18.4	135.1±40.2*

*P<0.05, compared with before; MVC – maximal voluntary contraction, CAR – central activation ratio; P1, P20, P100 – 1 Hz, 20 Hz, 100 Hz electrical stimulus, respectively; P1/P100 – 1/100 Hz ratio; HRT – Half-relaxation time. Fast cooling group (FC); Slow cooling group (SC). Values are means ± SD.

### Effects of body cooling on spinal and supraspinal reflex excitability

**Figure 2:**
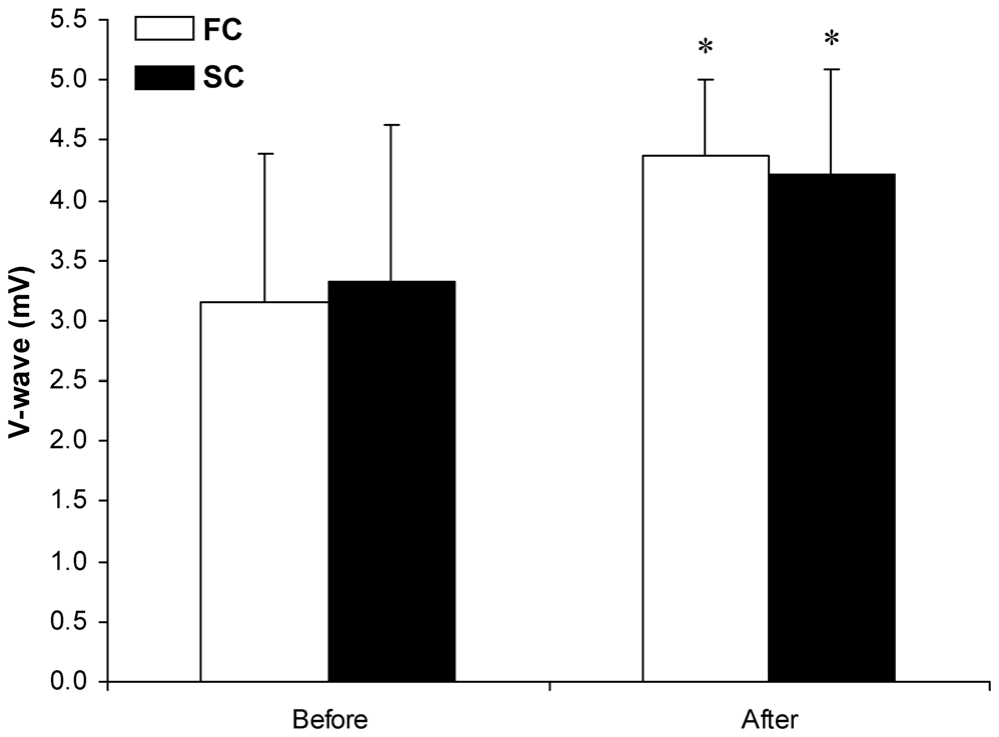
Changes in supraspinal (V-wave) response before and after body cooling. * P<0.05, compared with before. Fast cooling group (FC); Slow cooling group (SC).Values are means ± SD.

Whole body cooling increased the amplitude of H_max_-reflex (main time effect, *F*_1,38_ = 55.32, P<0.001, OP>99%) and H_max_/M_max_ ratio (main time effect, *F*_1,38_ = 55.88, P<0.001, OP>99%), and increased the latency time of H_max_-reflex (main time effect, *F*_1,38_ = 275.45, P<0.001, OP>99%) and M_max_-wave response (main time effect, *F*_1,38_ = 178.62, P<0.001, OP>99%); however, there were no significant differences between the FC and SC groups (Table 6). The increase in the V-wave response from before to after body cooling was significant (main time effect, *F*_1,38_ = 42.08, P<0.001, OP>99%; Fig. 2).

**Table 6:** Spinal reflex excitability before and after body cooling.

Amplitude (mV)					Latency (ms)			
FC		SC			FC		SC	
Before	After	Before	After		Before	After	Before	After
4.61±0.21	4.51±0.23	4.63±0.21	4.45±0.28	M_max_	13.9±1.72	18.4±3.41*	14.2±1.82	19.8±2.83*
3.28±1.1	4.33±0.67*	2.81±1.1	3.91±1.31*	H_max_	37.9±2.4	44.6±2.91*	37.5±2.2	44.1±3.1*
0.72±0.25	0.94±0.14*	0.65±0.34	0.89±0.28*	H_max_/M_max_				

*P<0.05, compared with before. Fast cooling group (FC); Slow cooling group (SC). Values are means ± SD.

### Effects of body cooling on cognitive performance

Body cooling had a significant effect (main time effect, *F*_1,38_ = 76.14, P<0.001, OP>99%) in both groups only on choice reaction time, although there was no significant difference between the FC and SC groups (Table 7).

**Table 7:** Cognitive performance before and after body cooling.

	FC		SC	
	Before	After	Before	After
UTS (odd/even, s)	0.564±0.047	0.662±0.093*	0.586±0.075	0.656±0.091*
FDST (mean digit span, no)	6.58±0.073	6.31±0.71	6.41±0.52	6.07±0.47
FCRM (correct sample match, no)	7.5±0.9	6.9±1.3	7.6±1.1	7.3±0.9

*P<0.05, compared with before. Fast cooling group (FC); Slow cooling group (SC); Unpredictable task switching (UTS); Forward digit-span task (FDST); Forced-choice recognition memory test (FCRM); Values are means ± SD.

### Blood variables before and after body cooling

The changes in the leucocytes (*F*_1,38_ = 14.15, P<0.01, OP>90%) and percentages of neutrophils (*F*_1,38_ = 41.89, P<0.001, OP>99%), lymphocytes (*F*_1,38_ = 33.45, P<0.001, OP>99%) and monocytes (*F*_1,38_ = 16.14, P<0.001, OP>95%) were significant only in the SC group (Table 8). The concentrations of cortisol (main time effect, *F*_1,38_ = 18.61, P<0.001, OP>95%), corticosterone (main time effect, *F*_1,38_ = 14.14, P<0.01, OP>70%), epinephrine (main time effect, *F*_1,38_ = 17.12, P<0.001, OP>95%) and norepinephrine (main time effect, *F*_1,38_ = 34.12, P<0.001, OP>99%) changed significantly after body cooling in both groups, but these changes did not differ between the two groups (P<0.05). IL-6 and TNF-α concentrations did not change significantly after body cooling in both groups.

**Table 8:** The blood variables before and after body cooling.

	FC		SC	
	Before	After	Before	After
Leucocytes, ^×^10^9^/L	6.4±1.1	7.9±1.1#	6.5±0.96	11.9±2.3*
Neutrophils, %	50.0±9.2	57.6±8.3#	47.8±9.3	73.9±5.6*
Lymphocytes, %	35.8±7.2	28.9±6.3#	39.7±8.9	19.9±7.6*
Monocytes, %	9.7±3.1	10.2±2.1#	10.1±1.8	6.9±1.7*
Cortisol, nmol/l	467.9±85.3	565.1±68.1*	476.9±82.3	586.3±74.1*
Corticosterone, nmol/l	47.9±40.2	29.1±14.1*	47.8±21.7	27.2±14.9*
IL-6, pg/ml	15.4±23.4	15.9±25.4	6.4±5.6	8.9±7.1
TNF-α, pg/ml	4.6±0.8	3.91±0.9	5.4±4.1	7.5±6.6
Epinephrine, ng/ml	2.3±1.6	4.4±0.8*	1.9±1.2	4.9±1.9*
Norepinephrine, ng/ml	6.7±3.5	41.3±19.3*	5. 8±2.9	49.5±17.2*

*P<0.05, compared with before;

# P<0.05, between fast cooling (FC) and slow cooling (SC) groups. Values are means ± SD.

### Correlation between indicators of cold stress

We found no significant correlations (r<0.15) between the CSI and changes in motor and cognitive performance, stress markers (cortisol, epinephrine and norepinephrine concentrations) or immune variables. A significant strong negative correlation was found between MHP and fat thickness: r = −0.49 and r = −0.53 in the FC and SC groups (P<0.05), respectively.

## Discussion

The main aim of this study was to compare the responses of motor and cognitive function, and stress indicators in the blood variables between people who exhibited fast or slow cooling responses to 14°C cold water immersion. The CSI was greater in the FC than in the SC group. However, contrary to our expectation, motor and cognitive performance did not differ between the FC and SC groups. In the SC group, whose CSI was lower, the cold stress seemed to stimulate some markers of innate (natural; for instance changes in neutrophils) immunity but suppressed markers of specific (adaptive; for instance changes in lymphocytes and monocytes) immunity. By contrast, the increases in the concentrations of cortisol, epinephrine and norepinephrine, as stress markers, did not differ between the FC and SC groups.

### Two strategies for response to 14°C cold water immersion

T_mu_ and T_sk_ did not differ between the FC and SC groups before and after cold exposure, whereas T_re_ decreased more and faster during cooling in the FC group. On acute exposure to cold, all subjects had an insulative response (a reduction in T_sk_ relative to T_re_), hypothermic response (a reduction in T_re_) and a metabolic response (an increase in heat production). The mean T_sk_ decreased significantly in both groups. During cold exposure, the body attempts to maintain a normal body temperature by increasing (by about 600%) MHP and by minimizing heat loss, primarily through shivering and peripheral vasoconstriction. These mechanisms can also induce hormonal and metabolic changes.

According to the definitions of Makinen [38], the response of the FC group to cooling vs the SC group response was more likely an insulative–hypothermic response, because the T_re_ decreased more in relation to changes in the T_sk_ and MHP compared with the SC group (T_sk_ decreased and MHP increased significantly in both groups, but there was no significant difference between the groups). The SC vs the FC group probably displayed a metabolic–insulative response, because the T_re_ decreased significantly less in relation to changes in T_sk_ and MHP than it did in the FC group. There are large individual differences in the relative contribution of the insulative response and the metabolic response to mild cold exposure. Therefore, the responses of the FC and SC groups to 14°C cold water immersion in this study probably reflect complex interactions and should be interpreted with some limitation. It is interesting to note that metabolic shivering was significantly greater in the FC than in the SC group, but subjective shivering did not differ between groups. We do not know whether the subjects displayed non-shivering thermogenesis during cold exposure, and we can only speculate that non-shivering thermogenesis may have been greater in the SC group, because this group displayed a smaller decrease in T_re_ and less metabolic shivering, but similar changes in T_sk_ compared with the FC group.

There is recent evidence that non-shivering thermogenesis (metabolic response strategy) by sympathetic, norepinephrine-induced mitochondrial heat production in brown adipose tissue is a component of this metabolic response in healthy men and that body fat content correlates negatively with brown adipose tissue content [65]. Our finding of a significant negative correlation between body fat mean thickness and MHP in both groups is consistent with this inverse relationship between body fat level and brown adipose tissue content. Body fat thickness did not differ significantly between the FC and SC groups in our study. As suggested by van Marken Lichtenbelt et al. [65], it is possible that some subjects could have had greater proportion of active brown adipose tissue, which could produce more heat during cooling. In our study, metabolic shivering was significantly greater in the FC than in the SC group, and we speculate that the SC group might have had more active brown adipose tissue.

### Despite the greater CSI in the FC group, the neuroendocrine response to cold stress did not differ between groups

This study was designed to examine the acute effects of intermittent cold exposure. Our cold exposure protocol does not represent the typical conditions that humans encounter during daily activities that might provoke a natural stress response. According to Kozyreva et al. [31], the FC group should have exhibited greater stress because FC induces more stress than does SC. However, contrary to our expectation, the stress markers in the blood (cortisol, epinephrine, norepinephrine and corticosterone concentrations) did not differ between the SC and FC groups. We had expected that the FC group with a higher CSI should have had greater activation of the sympathetic–adrenomedullary and hypothalamic–pituitary–adrenocortical systems, as generally occurs in response to stress [23]. These results should be interpreted with some limitation because the duration of cooling differed between the groups; i.e., immersion in cold water was nearly twice as long (170 min) in the SC group as in the FC group (96 min), and T_re_ decreased less in the SC group. We speculate that the stress markers in the SC group would have increased less if they had experienced the same duration of cold water exposure. We did not find significant differences in the perception of cold between the FC and SC groups.

### Immune variables changed significantly after cold stress only in the SC group

We expected that the immune system response to cold stress would be greater in the FC group because the CSI was greater. However, contrary to our expectation, markers of immunity changed significantly only in the SC group, which had a lower CSI. Cold exposure increased leucocyte count and neutrophil percentage and decreased lymphocyte and monocyte percentages only in the SC group; IL-6 and TNF-α concentrations did not change significantly in either group. This concurs with other research data showing that stress-induced changes in plasma corticosterone are accompanied by significant decreases in the numbers and percentages of lymphocytes and increases in the numbers and percentages of neutrophils. Our data suggest that cold exposure stimulated innate (natural) immunity and suppressed specific (adaptive) immunity only in the SC group. These data are consistent with the view that acute stressors are associated with potentially adaptive upregulation of some parameters of natural (innate) immunity and downregulation of some aspects of specific immunity. Specific immunity is characterized by greater specificity and a slower response than natural immunity [56]. However, the results of our study should be interpreted with some limitation because the immune response to cold stress depends on the cooling protocol [27], [33] and because not all markers of natural and specific immunity respond similarly (Table 8). For example, one marker of natural immunity (neutrophil percentage) increased significantly after cold exposure, whereas other markers such as IL-6 and TNF-α concentrations did not change.

Consistent with the results of human studies, in our study, acute cold stress increased circulating leucocyte and neutrophil counts. In contrast to Brenner et al. [8], we did not observe an increase in plasma IL-6 concentration in response to cold exposure. However, our results agree with those of Jansky et al. [27] who found a non-significant trend toward reduction in IL-6 concentration. Brenner et al. [8] reported positive relationships between changes in the concentrations of IL-6, norepinephrine and cortisol, and that the innate immune system was not adversely affected by brief cold exposure. They concluded that natural immunity is better suited than specific immunity to managing the potential complications of life-threatening situations, which can unfold more rapidly, because innate immunity is subject to fewer inhibitory constraints and requires less energy to be diverted from other bodily systems that support the fight-or-flight response [17].

Acute stress stimulates whereas chronic stress suppresses immune function [15]. The stress responses involve a variety of immune system hormones referred to as cytokines [30]. The most studied of these hormones with regard to stress are the pro-inflammatory cytokines IL-6 and TNF-α, which are involved in the acute-phase response to inflammatory stressors [30]. Recent evidence indicates that glucocorticoids and catecholamines, the major stress hormones, inhibit the production of pro-inflammatory cytokines such as IL-6 and TNF-α [20]. Charmandari et al. [10] concluded that IL-6 plays a major role in the overall control of inflammation by stimulating glucocorticoid secretion and by suppressing the secretion of TNF-α. Elevation of IL-6 concentration may increase symptoms of depression. Pro-inflammatory cytokines are signalling molecules of the inflammatory immune system that initiate and coordinate the cascade of immune events [20].

There is no current consensus about the response of the immune system to cold stress. Even brief exposure to cold increased the levels of norepinephrine, cortisol and lymphocytes [33]. Data obtained mainly on small mammals suggest that acute exposure to severe chilling suppresses several cellular and humoral components of the immune response [58]. Experiments on cell lines and in animals along with human experiments indicate that short-term (2–4-h) hypothermia increases the levels of anti-inflammatory cytokines and decreases the levels of pro-inflammatory cytokines [50]. We believe that further studies are needed to explain fully whether and to what extent cold exposure stimulates or suppresses the immune response.

### Motor and cognitive performance did not differ after cold stress between the FC and SC groups

We observed no significant difference in the changes in the electrically induced and voluntary properties of skeletal muscles and spinal and supraspinal reflexes after cooling between the FC and SC groups (Table 6). This was contrary to our expectations because we had expected that the larger CSI in the FC group would induce more changes in motor response. There is recent evidence that both voluntary and evoked-force development capacities of skeletal muscles are unimpaired until cooling is quite severe (<27°C) [18]. Although our subjects' muscle temperature did not decrease until 27°C, we found that P100 and HRT changed significantly after cold exposure in both the FC and SC groups. It has been suggested recently that stress also can modulate movement through activation of the hypothalamic–pituitary–adrenal axis (HPA-axis) and stress-associated emotional changes [41].

We also found that cold stress significantly increased the excitability of spinal (H-wave) and supraspinal (V-wave) reflexes in both groups but that there was no significant difference between the groups. These data agree with those of Dewhurst et al. [14] and Palmieri-Smith et al. [47], who showed that the H-wave and H_max_/M_max_ were facilitated after cold exposure. It was concluded that norepinephrine increases the response of motor neurons to their synaptic inputs and the excitability of interneurons that mediate group I input to motor neurons [25].

An excess of norepinephrine in the blood could contribute to the change in reflex excitability following body cooling. Because H_max_ is an indirect estimate of the number of motor neurons being recruited and M_max_ represents the entire motor neuron pool, the H_max_/M_max_ ratio can be interpreted as the proportion of the entire motor neuron pool capable of being recruited. We have not found any data in the literature about how cold stress influences the V-wave. The V-wave indicates the level of descending voluntary drive conveyed by the motor neurons, and an increase in the V-wave size indicates increased motor neuron discharge rates or recruitment [1]. However, according to Aagaard et al. [1], motor neuron discharge rate reflects both the supraspinal input into the motor neuron and the response to all inputs.

There is a clear effect of temperature on motor [22] and cognitive performance [46], which appears to follow an inverted U relationship. Stressful events can lead to immediate and marked impairments in working memory, which is an executive function [57]. There is evidence showing that the mechanisms and neural circuits that drive emotion and cognition are inextricably linked. There are two major components of the stress response: rapidly acting autonomic sympathetic system and the slower acting HPA-axis, which can affect cognitive functioning [28], [36], [37], [55]. Autonomic sympathetic nervous system responses include the release of the catecholamines epinephrine and norepinephrine from the adrenal medulla which causes the organism's ‘fight or flight’ response (e.g. increases the heart rate and blood pressure or enhances blood flow to skeletal muscles) [36], [64], [55]. Epinephrine can indirectly affect the brain through its action on vagus outside the brain-barrier with information transmitted in to the brain via nucleus of the solitary tract and locus coeruleus leading to the release of norepinephrine in the brain [28]. Activation of the HPA-axis as a result of stress or other causes of arousal initiates a flood of hormone and neurotransmitter release throughout the brain affecting the way we think, decide, and behave. Activation of the HPA-axis leads through intermediate steps to the release of glucocorticoid cortisol from the adrenal cortex [28], [36], [37], [64], [55], which travels through the bloodstream and accesses the brain where it binds to glucocorticoid receptors [28], [36], [37], [64], [55]. The prefrontal cortex, a brain region that governs higher-level cognitive processes and executive function, becomes markedly impaired by stress, producing measurable deficits in working memory [57]. Most of all cold stress had to affect unpredictable task switching of executive function because this task requires maintenance and additional processing (i.e., active manipulation of information) of the stored material by executive functions and more challenging cognitive tasks require more cognitive resources, which is more prone to be affected by stress [54]. Moreover, there is evidence of a dose–response relationship between decreased cognitive performance and core body temperature [46], suggesting greater deterioration of cognitive function with more marked core cooling. We failed, however, to find any other study showing the effect of cooling of different duration and different level of core body cooling on changes in the release of blood stress markers and cognitive functioning. In this study of the cognitive performance tests, cooling significantly affected only choice reaction time, however, contrary to our expectations, there was no difference between groups. Similar activation of autonomic sympathetic system and activation of HPA-axis might be expected here in both FC and SC groups because of similar release of stress markers in the blood found after the cooling and it might in part explain why there was no difference in cognition between groups. Specifically, the forward digit-span task and the forced-choice recognition memory test mainly involve the passive maintenance of information and require less cognitive resources [20], [21], [45], therefore, results of these tests did not change significantly in both groups during cold stress.

## Limitations and Perspectives

Our study had some limitations. The observations that an exposure time to 14°C cold water—which was nearly twice as short (96 min vs 170 min) with a greater T_re_ decrease (35.5°C vs 36.2°C) in the FC group compared with the SC group—induces similar responses of motor, cognitive, and blood stress markers were novel. This indicates that the SC group originally had a physiological advantage in prolonged exposure to an acute cold environment compared with the FC group. Nevertheless, the reasons for this remain unclear and we can only speculate on the effect of the same exposure time to 14°C cold water on motor, cognitive, and blood stress markers in the FC and SC groups. In this study, we found that immune variables changed from before to after cold exposure only in the SC group, which seems to be a more time-dependent variable than a T_re_-dependent variable. In line with the majority of thermal studies, we have used a protocol involving only two sampling time points to assess the responses of the HPA-axis, autonomic nervous system, and immune system to cold exposure. It is known, however, that in vitro mild hypothermia can suppress inflammatory reactions, and that moderate hypothermia can delay the induction of proinflammatory cytokines [29]. Therefore, in future studies, it will be important to examine the kinetics of the postcooling effect in FC and SC groups, as it seems that the analysis of a single time point at the end of cold stress does not cover the whole response curve.

## Conclusion

This study reports several new findings. Despite the larger CSI in the FC group the changes in stress markers (increases in cortisol, epinephrine and norepinephrine concentrations) did not differ between groups. The most important finding is that subjects with a lower CSI (SC group) showed stimulation of some markers of innate (natural) immunity and suppression of markers of specific (adaptive) immunity. However, contrary to our expectation, motor and cognitive performance response to cold stress did not differ between the FC and SC groups.
